# Intragastric Endoscopic Assisted Single Incision Surgery for Gastric Leiomyoma of the Esophagogastric Junction

**DOI:** 10.1155/2013/391430

**Published:** 2013-12-12

**Authors:** Salvador Morales-Conde, Isaias Alarcón, Carlos Ortiz-Moyano, Antonio Barranco, Francisco J. Padillo, María Socas

**Affiliations:** ^1^Unit of Innovation in Minimally Invasive Surgery, Department of General and Digestive Surgery, University Hospital “Virgen del Rocío”, Sevilla 41010, Spain; ^2^Unit of Gastroenterology, University Hospital “Virgen de Valme”, Sevilla 41010, Spain

## Abstract

Single port laparoscopic surgery is becoming an alternative to conventional laparoscopic surgery as a new approach where all the conventional ports are gathered in just one multichannel port through only one incision. Appling this technical development, we have developed a new technique based on an intragastric approach using a single port device assisted by endoscopy (I-EASI: intragastric endoscopic assisted single incision surgery) in order to remove benign gastric lesions and GIST tumors placed in the posterior wall of the stomach or close to the esophagogastric junction or the gastroduodenal junction. We present a patient with a submucosal gastric tumor placed near the esophagogastric junction removed with this new approach.

## 1. Introduction

Single port laparoscopic surgery is becoming an alternative to conventional laparoscopic approach in different procedures. The advantages of these techniques have still to be proven based on prospective randomized setting. But one of the main contributions of single port approach is a change in the mentality of those surgeons performing these techniques, since these new approaches open surgeons' minds to potential combined approach based on the concept of NOTES (natural orifice Transendoscopic surgery) and MANOS (minilaparoscopy assisted by natural orifice) or to an increase in the use of minilaparoscopic instruments. Hybrid procedures are increasing in number seeking a reduced injury of the anterior abdominal wall and also allowing surgeons and endoscopists to work together more and more in the operating room trying to reduce the invasiveness, thereby improving patient recovery. Old concepts such us intragastric laparoscopic surgery may be improved by the combination of single port access and endoscopic approach in order to offer new alternatives to different entities.

We have developed a new technique based on an intragastric approach using a single port device assisted by endoscopy (I-EASI: intragastric endoscopic assisted single incision surgery) in order to remove benign gastric lesions and GIST tumors placed in the posterior wall of the stomach or close to the esophagogastric junction or the gastro-duodenal junction.

## 2. Case Report 

A 40-year-old woman with a clinical history of persistent epigastric pain was diagnosed by gastroscopy of a gastric submucosal tumor in the esophagogastric junction. Endoscopic ultrasound showed a 20 mm submucosal tumor with benign features. The biopsy performed guided by the endoscopic ultrasound revealed a leiomyoma. The locations of the lesion lead us to perform a laparoscopic intragastric surgery in order to avoid a transgastric approach through a large opening over the anterior wall of the stomach or a large resection of the upper third of the stomach. This intragastric approach was performed by using a single port device placed at the right upper quadrant of the patient.

**Figure 1:**
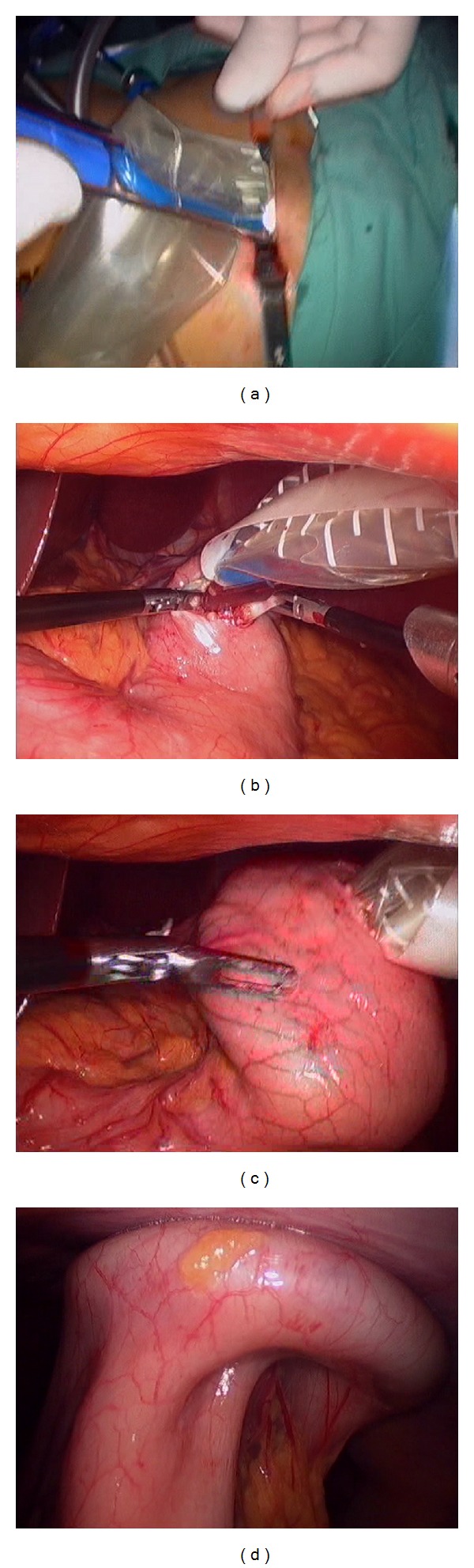
(a) Introduction of TriPort through abdominal wall. (b) Introducer is passed through gastrotomy. (c) Retraction of the device rising gastric wall up to abdominal wall. (d) TriPort fixed completely sealing both abdominal and gastric walls.

A conventional laparoscopy was performed using three 5 mm trocars, one supraumbilical trocar, for a 5 mm 30° optic, and two ports as working channels, being inserted in the left and the right upper quadrants. Once peritoneal cavity was preliminarily examined, a gastroscopy was performed to confirm the location of the lesion at the esophagogastric junction. A 1.5 cm gastrotomy was performed at the anterior wall of the stomach, far from the lesion, using the Harmonic scalpel (Ethicon Endo Surgery, Cincinnati, OH, USA). By enlarging to 1.5 cm the previous 5 mm incision of the right upper quadrant port, the introducer of a single port multichannel, TriPort Plus (Advanced Surgical Concepts, Ireland) was introduced under laparoscopic vision through the abdominal wall into the gastrotomy. The self-expanding shaft of the TriPort Plus helps the device to firmly grip both abdominal and stomach walls allowing CO_2_ insufflation of the stomach (Figure 1).

**Figure 2:**
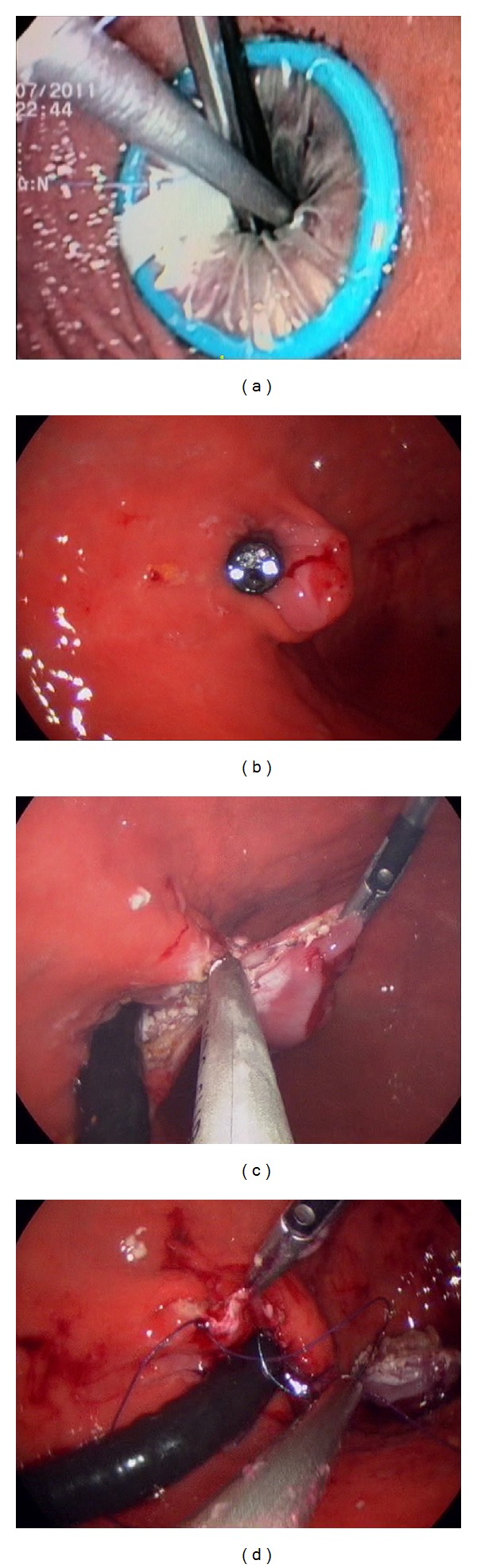
(a) Intragastric endoscopic vision of laparoscopic devices through Triport. (b) Location of tumor at esophagogastric junction. (c) Resection of tumor assisted by traction of the cardias performed with the gastroscope. (d) Closure of mucosal defect.

**Figure 3:**
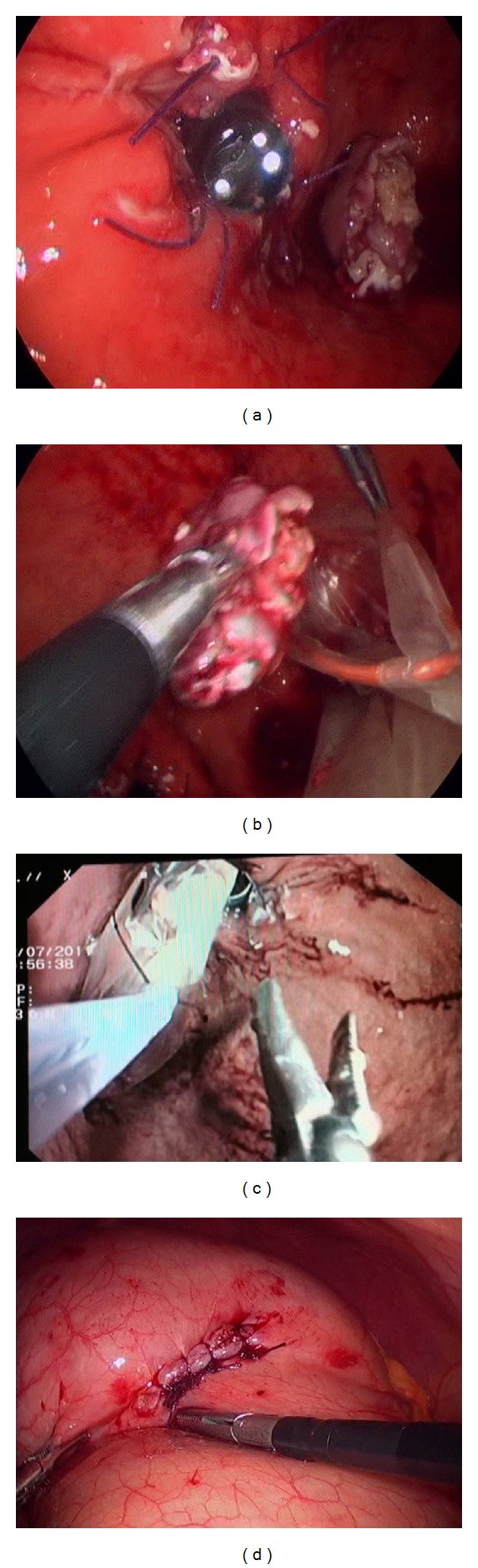
(a) Confirmation of good passage through esophagogastric junction after closure with endoscope. (b) Introduction of tumor in endobag. (c) Tumor removal through gastroscope. (d) Closure of gastrotomy.

The 5 mm 30° optic, a roticulator atraumatic grasper (Covidien, Mansfield, MA, USA), and standard laparoscopic instruments were introduced through the single port device in order to work inside the stomach. Tumor location at esophagogastric junction was confirmed. Tumor was resected using the Harmonic scalpel, respecting the muscle layer of the esophagogastric junction. Simultaneously endoscopic movements of the scope provide us a good traction of the cardias. The mucosal defect left behind was repaired with intracorporeal interrupted absorbable sutures (Figure 2). A good passage through esophagogastric junction was checked by removing and introducing the gastroscope once defect was repaired. An endobag was introduced through the single port device in order to introduce the specimen, being removed by the endoscopist inside the bag through the mouth of the patient. The TriPort Plus was removed from the stomach and placed just in the abdominal wall and the gastrotomy was closed using four absorbable sutures. The stomach was insufflated by the endoscopist and no leaks were observed at the anterior gastric wall (Figure 3).

Patient was discharged home on day one postoperatively. Three months after surgery the patient remained asymptomatic with no side effect after this approach. Histological examination of the surgical specimen confirmed the presence of a leiomyoma.

## 3. Discussion

Laparoscopic resection of benign gastric submucosal tumors may be performed by using three types of approaches: exogastric, transgastric, and intragastric [1]. Exogastric laparoscopic resection is carried out without opening the gastric cavity and it is useful for those tumors located at the anterior wall, lesser curvature, and antrum. This technique resects the tumor and closes the gastrotomy simultaneously by sequential application of the different cartridges of an endostapler. But for those tumors located close to the esophagogastric junction, the pylorus, or at the posterior wall of the stomach, laparoscopic exogastric resection is difficult to be performed [2] and might be related to potential complications such as deformity or stenosis [3].

For those “difficult” settings, transgastric laparoscopic approach provides a proper visualization of the lesions and an accurate location of tumors with intraluminal growth that cannot be reached by exogastric approach [4]. The surgical approach of these lesions requires an extended gastrotomy at the anterior gastric wall in order to allow a large surgical field to introduce the instruments required for the resection. Nevertheless, this gastrotomy may be related to potential complications as bleeding or peritoneal contamination [2].

Laparoscopic intragastric surgery (LIGS), described by Ohashi [5], consists in a direct introduction of the laparoscopic ports into the gastric cavity through the anterior gastric wall, improving the visualization and the operative field, while minimizing the damage of the gastric wall and facilitating the closure of the stomach. LIGS was initially described using one 10 mm port for the optic and two 5 mm working ports inserted through gastric wall. The combination of endoscopic and laparoscopic intragastric approach eases the technique, avoiding the introduction of extra assistant ports through the gastric wall and facilitating the insertion of intragastric trocars by distending the stomach properly creating a “pneumostomach” with the air insufflated by the endoscopist. But the introduction of the ports is one of the handicaps of this technique. The creation of the pneumostomach requires duodenum clamping, due to air migration to small bowel causing intestinal distension in the postoperative period [1]. The use of carbon dioxide (CO_2_) to create this pneumostomach minimizes this complication as CO_2_ is absorbed faster than air and no duodenal clamping is needed [6]. On the other hand, to maintain the position of the intragastric ports, specially designed ports with self-retaining devices, such a balloon, must to be used to keep the abdominal wall ports inside the gastric cavity. Another limitation related to LIGS is the retrieval of the specimen, as large tumors cannot be extracted through gastric ports and must be removed orally or by enlarging one of the ports.

The advantages of the technique described by our group (I-EASI) allow just one incision at the anterior gastric wall instead of the multiple punctures needed in conventional LIGS and no especial trocars are needed. This multiport device allows the use of up to three instruments, besides the hole of the device used for the camara. Self-adjusting ability of TriPort helps us to maintain the port in an intragastric position and no leaks of the pneumostomach. In addition, we can introduce the TriPort device without a distended stomach, creating the “pneumostomach” once the TriPort is fixed and sealed to the abdominal wall, what reduces the amount of air insufflated, what could be related to less discomfort in the postoperative period. Furthermore, specially designed TriPort allows us to retrieve larger specimens than standard trocars during conventional laparoscopic intragastric surgery, avoiding oral extraction or gastrotomy, although in this case the specimen was removed through the mouth. On the other hand, this technique maintains the advantages of endoscopic assisted procedures since endoscopist may assist the procedure with the pneumostomach maintain with CO_2_ insufflated through the single post device.

With the current experience gathered on single port access surgery in our group, we think that there are no technical restrictions compared with conventional laparoscopic surgery to remove this type of lesions, and single port intragastric surgery using TriPort Plus (I-EASI) presents some advantages regarding conventional LIGS.
